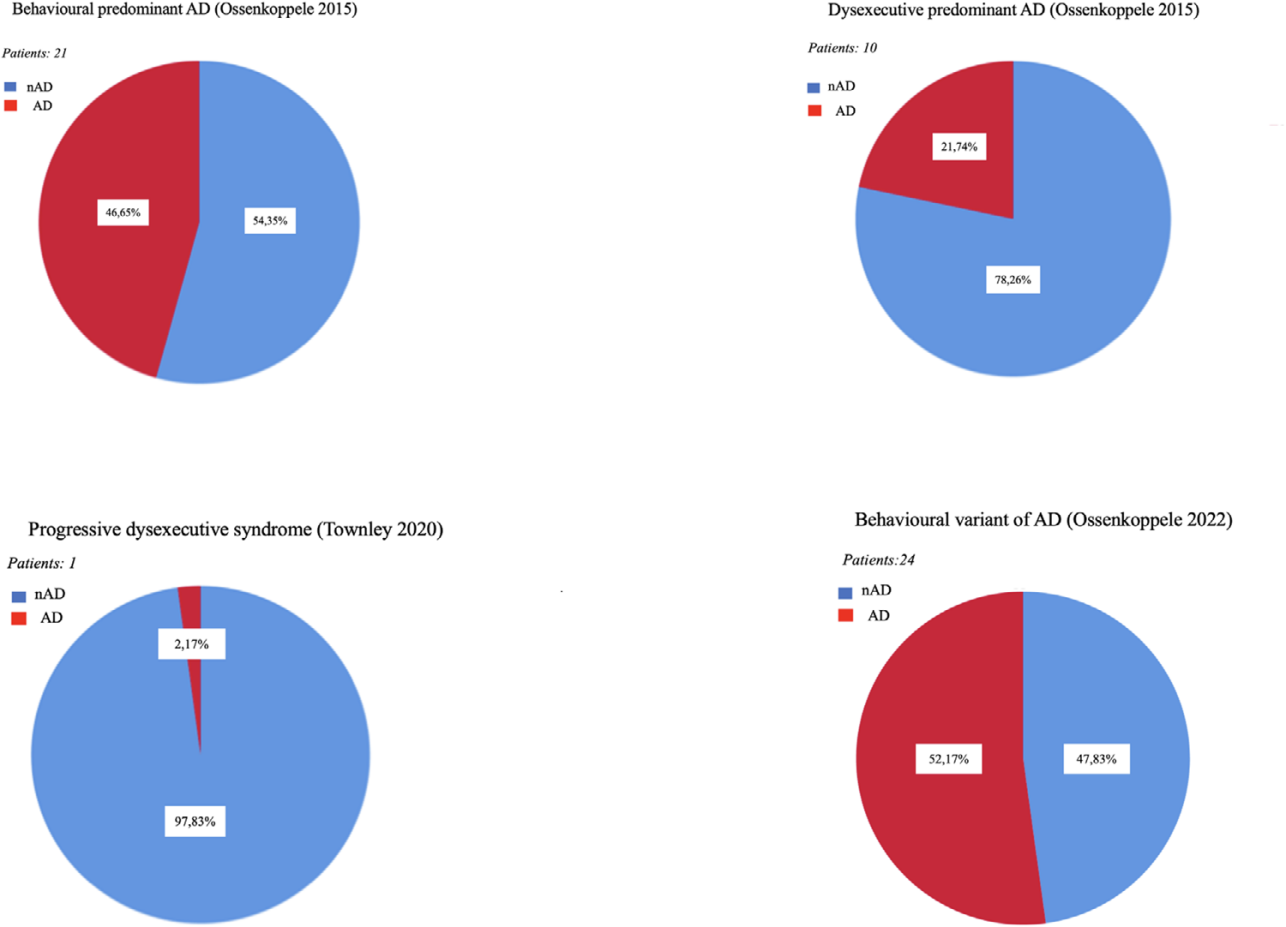# Accuracy of clinical diagnostic criteria for behavioral/dysexecutive Alzheimer’s Disease in patients presenting with mild behavioural or dysexecutive symptoms

**DOI:** 10.1002/alz.092441

**Published:** 2025-01-03

**Authors:** Najara Iacovino, Manuela Tondelli, Chiara Carbone, Chiara Gallingani, Maria Angela Molinari, Annalisa Chiari, Giovanna Zamboni

**Affiliations:** ^1^ Università di Modena e Reggio Emilia, Modena Italy; ^2^ Neurologia, Azienda Ospedaliero Universitaria di Modena, Modena Italy; ^3^ Dipartimento di Cure Primarie, AUSL Modena, Modena Italy

## Abstract

**Background:**

Diagnosis in patients with Mild Behavioural Impairment (MBI) and with Mild Cognitive Impairment (MCI) with predominant executive deficits (eMCI) is often challenging, as they may be representing the early phase of both Alzheimer’s Dementia (AD) as well as behavioural variant Frontotemporal Dementia (bvFTD). If neuropathology biomarkers aren’t available, diagnosis is even more difficult. We evaluated the performance of classification of different clinical diagnostic criteria for behavioural/dysexecutive AD (without biomarkers) in MBI and eMCI.

**Method:**

Consecutive patients with a clinical diagnosis of MBI or eMCI seen in our Cognitive Neurology Clinic from January 2022 to December 2023 were retrospectively recruited, irrespectively of the presence of other cognitive signs. To be included they needed to have undergone measurement of biomarkers of amyloidosis and neurodegeneration (p‐tau, t‐tau, Abeta42‐40 ratio in cerebrospinal fluid or (^18^F) Flutemetamol‐PET) and extended neuropsychological evaluation. Blind to biomarkers, we classified patients according to the following different clinical criteria: (i) Behavioural or Dysexecutive predominant AD (Ossenkoppele 2015), (ii) Progressive dysexecutive syndrome (Townley 2020), and (iii) behavioural variant of AD (bvAD) (Ossenkoppele 2022). Diagnostic accuracy of each criterion was then compared to biomarkers’ classification for differentiating AD (positive biomarkers) from bvFTD continuum (negative biomarkers). Sensibility and specificity were determined for each criterion and Kappa coefficient used for assessing agreement between different sets of criteria.

**Result:**

Forty‐six consecutive eligible patients were included. Among them, 45.6% fulfilled criteria for Behavioural predominant AD, 21.7% for Dysexecutive predominant AD, 2.1% for Progressive dysexecutive syndrome, and 52.1% for bvAD. Diagnostic accuracy analyses using biomarkers showed that criteria for Behavioural predominant AD correctly classified 78.2% of patients (sensibility 77.7%, specificity 78.9%), criteria for Dysexecutive predominant AD 67.3% (sensibility 88.8%, specificity 38.8%), criteria for Progressive dysexecutive syndrome 60%, and bvAD criteria 80.4% of patients (sensibility 74%, specificity 89.4%). Higher kappa coefficient (k = 0.85) was found between Behavioural‐predominant AD and bvAD criteria.

**Conclusion:**

Clinical criteria to diagnose patients presenting with mild behavioural or dysexecutive symptoms have poor diagnostic accuracy in absence of biomarkers measurement. Among the criteria available, the definition of bvAD proposed by Ossenkoppele (2022) achieves the more accurate performance in distinguishing AD from bvFTD continuum.